# Tranilast Modulates Cellular and Matrix Remodeling in Achilles Tendon Healing: A Comprehensive Histological and Biomechanical Analysis

**DOI:** 10.3390/bioengineering13070768

**Published:** 2026-06-30

**Authors:** Oktay Adanır, Ozancan Bicer, Yigit Guleryuz, Muhammed Uslu, Abdurrahman Acar, Busra Yaprak Bayrak

**Affiliations:** 1 Department of Orthopedics and Traumatology, Bagcilar Training and Research Hospital, University of Health Sciences, Istanbul 34200, Turkey; 2 Department of Orthopedics and Traumatology, Beykoz State Hospital, Istanbul 34800, Turkey; 3 Department of Orthopedics and Traumatology, Pendik Training and Research Hospital, Marmara University, Istanbul 34899, Turkey; 4 Department of Pathology, School of Medicine, Kocaeli University, Kocaeli 41380, Turkey

**Keywords:** tranilast, Achilles tendon, tendon healing, Bonar score, Movin score, biomechanics, collagen, rat model

## Abstract

Background: Tranilast is an anti-inflammatory and antifibrotic agent known to inhibit TGF-β-mediated fibroblast activation and matrix overproduction. Although its therapeutic potential has been explored in several fibrotic and inflammatory conditions, its effects on tendon healing remain unknown. This study aimed to evaluate the histopathological and biomechanical impact of tranilast in a rat Achilles tendon repair model. Methods: Thirty-two male Sprague–Dawley rats were randomly assigned to four subgroups, control (C-15, C-30) and tranilast-treated (TR-15, TR-30), and sacrificed on postoperative day 15 or 30. Tranilast (30 mg/kg/day) or placebo was administered intraperitoneally. Tendons were subjected to biomechanical testing (maximum load to failure) and histological evaluation using Bonar and Movin scoring systems on Hematoxylin + Eosin (HE)-, Masson Trichrome (MT)- and Alcian Blue (AB)-stained slides. Additional analyses included Sirius Red histochemistry with assessment of collagen type I/III intensity and polarization ratio, as well as H-scores for collagen I and III. Results: Tranilast produced marked improvements in histopathological healing. Both TR-15 and TR-30 exhibited significantly lower Bonar and Movin scores compared with controls, with reductions in tenocyte degeneration, ground-substance accumulation, collagen disorganization, vascularity, and hyalinization (all *p* < 0.01). However, biomechanical strength did not differ significantly among the groups (*p* = 0.3948). Sirius Red analysis and collagen I/III H-scores revealed no significant differences in collagen composition or polarization ratio. Histological improvements were therefore not accompanied by measurable changes in collagen subtype distribution or maximum load to failure. Conclusions: Tranilast was associated with favorable histopathological changes during Achilles tendon healing, including improvements in cellular morphology, matrix organization, and overall Bonar and Movin scores. However, these findings were not accompanied by significant differences in collagen composition or biomechanical strength within the 30-day observation period. Further long-term and mechanistic studies are warranted to determine whether these histopathological changes translate into functional improvements in tendon biomechanics. Accordingly, the present findings should be considered preliminary and exploratory.

## 1. Introduction

Tendon healing is a complex biological process involving inflammation, proliferation, and remodeling phases, which ultimately aim to restore tissue continuity and mechanical strength [1,2]. However, excessive fibroblast activation and collagen deposition often result in scar tissue formation, leading to reduced tendon elasticity and mechanical performance [3,4,5]. Therefore, the modulation of fibrosis represents a crucial therapeutic target in tendon repair. Various antifibrotic agents have been investigated to limit excessive extracellular matrix deposition in musculoskeletal tissues [5,6,7,8,9].

Among these agents, Tranilast (N-[3′,4′-dimethoxycinnamoyl]-anthranilic acid) has attracted considerable attention. Initially developed as an antiallergic compound, tranilast has been approved for clinical use in Japan and South Korea since 1982 for the treatment of bronchial asthma, atopic dermatitis, and allergic rhinitis [10,11,12,13,14,15]. Chemically, Tranilast is an analog of a tryptophan metabolite and has shown a favorable safety profile with minimal adverse effects in humans [10,11,12,14]. Beyond its antiallergic activity, extensive research has revealed that Tranilast possesses broad anti-inflammatory and antifibrotic effects, expanding its therapeutic potential to numerous pathological conditions including fibrotic diseases, proliferative disorders, cardiovascular and autoimmune diseases, ocular pathology, diabetes, renal diseases, and several forms of cancer [16].

The antifibrotic properties of Tranilast were first described by Suzawa et al. (1992), who demonstrated that the drug inhibited the release of profibrotic cytokines from monocyte–macrophage lineages in vitro [17]. Subsequent experimental studies reported antifibrotic effects in multiple tissues, including diabetic renal fibrosis, cardiac fibrosis, and skeletal muscle fibrosis, and suggested that Tranilast may suppress TGF-β-related profibrotic signaling pathways [18,19,20,21,22,23,24].

The reported antifibrotic actions of Tranilast have been linked to modulation of profibrotic signaling, including TGF-β-related pathways, together with broader effects on inflammatory and fibrotic mediators. Collectively, the available evidence suggests that Tranilast may reduce fibroblast proliferation, collagen synthesis, and inflammatory cytokine release, thereby limiting tissue fibrosis in a variety of experimental settings [6,24,25].

Achilles tendon pathology is clinically important because even when tendon continuity is restored, patients may experience prolonged functional limitations and variable recovery trajectories, and minimally invasive/percutaneous repair strategies continue to evolve [26,27,28,29,30,31]. Despite growing evidence of Tranilast’s antifibrotic efficacy in multiple organ systems, its potential influence on tendon healing remains unexplored. Given the pivotal role of fibroblast-mediated collagen remodeling in tendon repair, elucidating the effects of Tranilast on tendon regeneration may provide novel insights into antifibrotic therapeutic strategies. Therefore, this study aimed to investigate the histopathological and biomechanical effects of Tranilast following surgical repair of the rat Achilles tendon. Because the selected time points primarily represent the proliferative and early remodeling phases of healing, the findings should be interpreted within the context of early reparative responses.

## 2. Materials and Methods

### 2.1. Experimental Design and Animals

This experimental study included 32 young adult male Sprague–Dawley rats (aged 12–14 weeks, weighing approximately 330 g at the time of surgery). Male rats were exclusively used to minimize potential variability related to hormonal fluctuations associated with the estrous cycle, which has been reported to influence tendon biology, collagen turnover, and inflammatory responses. This approach is commonly adopted in experimental tendon healing models to ensure greater homogeneity and reproducibility of histopathological and biomechanical outcomes.

**Figure 1:**
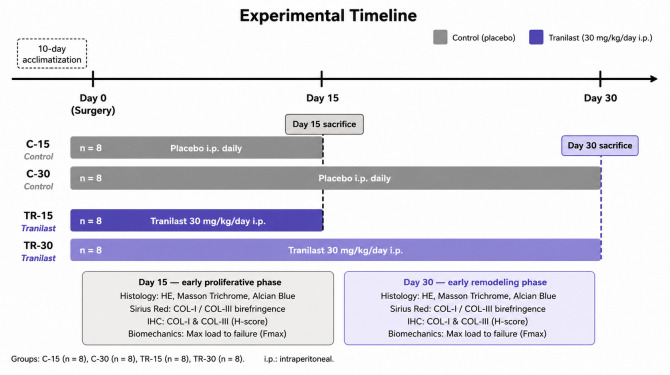
Experimental timeline. After 10 days of acclimatization, 32 male Sprague–Dawley rats underwent Achilles tendon transection and repair (Day 0) and were randomly assigned to four subgroups (n = 4/subgroup for histology; n = 4/subgroup for biomechanics): control groups that received daily intraperitoneal placebo injections (C-15, C-30) and tranilast-treated groups that received 30 mg/kg/day tranilast intraperitoneally (TR-15, TR-30). Animals were sacrificed on Day 15 (early proliferative phase) or Day 30 (early remodeling phase). Harvested tendons were analyzed by HE, Masson Trichrome, and Alcian Blue staining (Bonar and Movin scoring), Sirius Red histochemistry (collagen type I/III birefringence), collagen type I and III immunohistochemistry (H-score), and biomechanical testing (maximum load to failure, Fmax). C, control; TR, tranilast; i.p., intraperitoneally.

The animals were randomly divided into two main groups (n = 16 each): a Control group (C) and a Tranilast group (TR). Each group was further subdivided for sacrifice at either 15 day (early reparative/proliferative phase) or 30 days (early remodeling phase) after tendon repair, yielding four subgroups (Figure 1):

• C-15: Control group, sacrificed at day 15 (after daily intraperitoneal administration of 1 mL of distilled water as placebo);
• C-30: Control group, sacrificed at day 30 (after daily intraperitoneal administration of 1 mL of distilled water as placebo);
• TR-15: Tranilast group, sacrificed at day 15 after daily intraperitoneal administration of Tranilast (30 mg/kg/day);
• TR-30: Tranilast group, sacrificed at day 30 after daily intraperitoneal administration of Tranilast (30 mg/kg/day).

Tranilast (T714570, Toronto Research Chemicals, Toronto, ON, USA) was administered intraperitoneally once daily. Intraperitoneal administration was selected to ensure consistent systemic exposure and dosing accuracy in a small-animal model while avoiding variability related to local peritendinous distribution, leakage, or injection-site effects. This systemic route is widely used in rodent pharmacology studies where stable daily exposure is required. The dose (30 mg/kg/day) was selected based on prior in vivo studies demonstrating anti-inflammatory/antifibrotic efficacy of systemic Tranilast in rodent models, and was chosen at the lower end of commonly used ranges to balance pharmacologic activity with tolerability [16,24]. Control animals received the same volume of distilled water under identical conditions.

### 2.2. Housing and Husbandry

All animals were obtained from the Experimental Research and Skill Development Center (BADABEM), Local Health Application and Research Center, which is an accredited institutional laboratory animal breeding and research facility.

All animals were housed in the Experimental Animal Laboratory under controlled environmental conditions (temperature: 22 ± 2 °C, humidity: 40–60%) with a 12 h light/12 h dark cycle. Standard pellet chow and tap water were provided ad libitum. Rats were acclimatized for 10 days prior to the experiment. They were then allocated into four subgroups (C-15, C-30, TR-15, TR-30) and housed in different cages, each containing 4 rats, with proper labeling. Body weights were recorded weekly throughout the 4-week experimental period.

### 2.3. Surgical Procedure

A standardized Achilles tendon rupture and repair model was created; all operations were performed by the same surgeon to minimize variability. Each rat was weighed preoperatively to calculate the precise drug doses. All animals received a single preoperative subcutaneous injection of gentamicin (8 mg/kg) for antibiotic prophylaxis and were anesthetized with ketamine/xylazine (80/10 mg/kg, intramuscular). The right hind limb was shaved and disinfected with povidone-iodine (Batticon^®^, Adeka, Samsun, Türkiye), and the operative area was covered with sterile drapes. A 2 cm longitudinal incision was made on the posterior aspect of the right ankle to expose the Achilles tendon. A complete transverse tenotomy was performed 2–4 mm proximal to the calcaneal insertion using a No. 11 scalpel blade. The tendon was repaired using the Kessler technique with 0-2 PDS sutures (DemeDiox^®^, DemeTECH, Miami Lakes, FL, USA). The skin was closed with 2-0 silk sutures (Sterisilk^®^, İstanbul, Türkiye), and the wound was cleaned with povidone-iodine.

For postoperative analgesia, carprofen (3 mg/kg; Rimadyl^®^, Pfizer, İstanbul, Türkiye) was administered subcutaneously. Rats were monitored individually for 24 h and returned to their cages thereafter. Analgesia was repeated every 12 h during the first postoperative day if required. No immobilization or movement restriction was applied. All animals were monitored daily for wound integrity, gait abnormalities, and signs of re-rupture or infection. Tendon integrity was assessed clinically by observing weight-bearing and gait function, and the repair site was inspected for abnormal gap formation, excessive swelling, or loss of continuity. In addition, at the time of harvest, all tendons were macroscopically examined to confirm preservation of the repaired tendon structure before biomechanical or histological processing. No postoperative complications, tendon re-ruptures, wound dehiscence, or exclusions occurred in any group during the follow-up period.

### 2.4. Treatment and Sacrifice Protocol

During the postoperative period, daily intraperitoneal injection was performed as follows:

• Control groups (C-15, C-30): 1 mL of distilled water (placebo);
• Tranilast groups (TR-15, TR-30): 30 mg/kg/day Tranilast in 1 mL of distilled water.

In both control subgroups (C-15 and C-30), animals received daily intraperitoneal placebo injections (1 mL distilled water) until sacrifice (day 15 or day 30, respectively). Animals in the C-15 and TR-15 groups were sacrificed on postoperative day 15 to evaluate early-reparative/proliferative-phase tendon healing, whereas animals in the C-30 and TR-30 groups were sacrificed on postoperative day 30 to evaluate early-remodeling-phase tendon healing. The selected time points were designed to capture two biologically distinct stages of tendon healing in rats: day 15 represents the early reparative/proliferative response with active cellularity and matrix turnover, and day 30 reflects a more advanced remodeling stage in which collagen organization and tissue maturation become more prominent.

From each subgroup, animals were randomly allocated for histomorphometric or biomechanical analyses, with four animals per subgroup used for histological evaluation and four animals per subgroup used for biomechanical testing (total n = 8 animals per subgroup; total n = 32 animals overall). Animals designated for biomechanical testing had both right and left Achilles tendons excised. The contralateral (left) intact Achilles tendon was collected as an internal reference to document native tendon mechanical behavior under the same testing conditions and to support quality control of specimen handling and clamping. Group comparisons, however, were performed using the repaired right tendons, which represented the experimental endpoint.

At each predetermined endpoint (days 15 and 30), animals were deeply anesthetized with an intraperitoneal overdose of ketamine (80 mg/kg) and xylazine (10 mg/kg). After confirming complete loss of the pedal reflex, humane euthanasia was performed by cervical dislocation in accordance with AVMA Guidelines (2020).

### 2.5. Histological, Histochemical, Immunohistochemical, and Biomechanical Analyses

**Figure 2:**
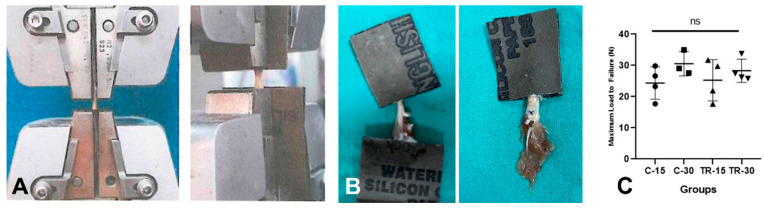
Biomechanical testing setup and maximum load to failure values. (**A**) Representative photographs of the electromechanical tensile testing machine (AVSA 500 kg/n) showing tendon specimen fixation between the clamps prior to testing (left: frontal view; right: lateral view). (**B**) Representative photographs of tendon-bone construct specimens following failure, demonstrating the rupture site at the repair zone. (**C**) Maximum load to failure (Fmax) values across the four experimental groups. Data are presented as scatter plots with individual data points superimposed on the mean ± standard deviation (SD; n = 4 per group). Each symbol represents an individual animal. No statistically significant differences were detected among groups (one-way ANOVA, *p* = 0.3948). C-15, control group sacrificed on postoperative day 15; C-30, control group sacrificed on postoperative day 30; TR-15, tranilast-treated group sacrificed on postoperative day 15; TR-30, tranilast-treated group sacrificed on postoperative day 30; ns, not significant.

Excised Achilles tendons, including both origin and insertion sites, were processed for either histological or biomechanical assessment. Samples for biomechanical testing were stored at −20 °C until analysis. Biomechanical tests were performed at the Yıldız Technical University Department of Mechanical Engineering Laboratory using an AVSA 500 kg/n electromechanical tensile testing machine (Instron 5982. Norwood, MA, USA) at a traction rate of 1 mm/s. The maximum load to failure (Fmax) was recorded digitally. The repair was performed using absorbable 0–2 PDS sutures, and sutures were not removed prior to mechanical testing; thus, the measured load-to-failure reflects the in situ tendon-repair construct at the time of harvest (Figure 2A,B).

For histological evaluation, specimens were fixed in 10% neutral buffered formalin, processed routinely, embedded in paraffin, and sectioned at 4 μm thickness. Slides were stained with hematoxylin–eosin (H&E) for morphological assessment. For histochemical evaluation, serial sections on positively charged slides were stained with Masson Trichrome (MT) and Alcian Blue (AB) using an automated histostainer and assessed according to the Bonar and Movin scoring systems. Additional sections were subjected to Sirius Red histochemical staining to assess collagen birefringence and relative collagen fiber composition using an automated immunostainer. Immunohistochemical staining for collagen type I and type III was performed using specific primary antibodies, and expression levels were quantified using an H-score method. Sirius Red staining was used as a complementary histochemical method to assess collagen organization and birefringence, rather than as a substitute for immunohistochemistry. Sirius Red-stained sections were examined under both bright-field and polarized light microscopy.

Quantitative measurements reported in Table 1 were obtained exclusively from digitally captured bright-field images using standardized image analysis protocols (intensity-based quantification). Polarized light microscopy was used for qualitative assessment of collagen birefringence at the microscope; however, representative polarized micrographs could not be digitally recorded because the available imaging system lacked a compatible polarized digital acquisition setup at the time of evaluation. Accordingly, no quantitative data were derived from polarized light observations, and all reported numerical values reflect objective image-based measurements rather than visual estimation.

Slides were evaluated under an Olympus CX51 microscope (Olympus Corp., Tokyo, Japan) by a pathologist blinded to group allocation and treatment status. All Bonar and Movin scores were assigned by the same blinded evaluator using predefined scoring criteria and randomized specimen assessment to minimize observer bias. Because scoring was performed by a single evaluator, inter-observer reliability and formal repeatability analyses were not assessed and should be addressed in future studies.

In Bonar scoring, four variables—tenocyte morphology/proliferation, ground substance (GAG) content, collagen bundle alignment, and vascularity—were each graded from 0 (best) to 3 (worst), yielding a total score of 0–12. For each Bonar parameter, grading was performed according to well-established histomorphological definitions described in the original Bonar scoring system, with score 0 representing near-normal tendon morphology and score 3 indicating severe pathological alteration. These definitions include progressive changes in tenocyte morphology and density, increasing ground substance accumulation, loss of parallel collagen bundle alignment, and increasing vascularity. All scores were assigned based on representative fields reflecting the dominant morphology of each specimen. In Movin scoring, eight parameters—fiber structure, fiber arrangement, nuclear morphology, regional cellularity, increased vascularity, decreased collagen staining (MT), hyalinization, and GAG content (Alcian Blue)—were each scored 0–3 (total = 24).

Immunohistochemical staining intensity for Collagen Type I and Type III was semi-quantitatively evaluated using an H-Score (Intensity × %Area; 0–300). This approach provides a standardized semi-quantitative measure of protein expression distribution across the tissue. The polarization ratio [Type I/(Type I + Type III)] was also calculated to assess tendon maturation.

Although both Bonar and Movin systems include a ground substance/GAG domain, the scoring definitions and the histochemical readout used to represent this domain are not identical; therefore, small differences in ordinal GAG trends between systems may occur, particularly in the presence of focal matrix heterogeneity.

Immunohistochemical staining intensities for Collagen Type I and Collagen Type III were semi-quantitatively assessed using the H-score method, which combines staining intensity and the percentage of positively stained tissue area to provide an integrated assessment of protein expression. The H-score was calculated as the product of staining intensity and the percentage of positive staining (range: 0–300), consistent with previously published tendon-healing studies. Additionally, the polarization ratio, calculated as Type I/(Type I + Type III), was used as an indicator of relative tendon maturation.

### 2.6. Statistical Analysis

All statistical analyses were performed using GraphPad Prism (version 8.1.1, GraphPad Software, San Diego, CA, USA). Data normality was assessed with the Kolmogorov–Smirnov test. Parametric variables were compared using one-way ANOVA, followed by Tukey’s multiple comparison test. Non-parametric data (histopathological scores) were analyzed using the Kruskal–Wallis test, and significant results were further evaluated with Mann–Whitney U tests for pairwise comparisons. Results were expressed as mean ± standard deviation (SD) or median [IQR], and a *p* < 0.05 was considered statistically significant. Histopathological scores (Bonar and Movin), being ordinal variables, were primarily analyzed using non-parametric methods; although these data are shown as mean ± SD for consistency with previous literature, they should be interpreted as ordinal semi-quantitative variables.

Given the exploratory nature of this experimental study, a formal a priori power analysis was not performed. Sample sizes were determined based on previously published experimental tendon-healing studies employing similar histopathological and biomechanical endpoints. Because each subgroup was divided into n = 4 specimens for histological evaluation and n = 4 specimens for biomechanical testing, the biomechanical analyses should be interpreted as exploratory and may have been underpowered to detect small-to-moderate treatment effects in the presence of biological variability.

A sensitivity-based power consideration indicated that, assuming α = 0.05 and 80% power, the available biomechanical sample size would be sufficient to detect only very large between-group effects for maximum load to failure (approximately Cohen’s f ≥ 0.97 for one-way ANOVA, corresponding to Cohen’s d ≥ 2.38 for pairwise comparisons). Therefore, smaller but potentially biologically relevant biomechanical differences may have remained undetected, and negative biomechanical findings should be interpreted with caution.

### 2.7. Ethical Approval

All animal experiments were conducted in accordance with the National Guidelines for the Care and Use of Laboratory Animals and complied with the EU Directive 2010/63/EU [32] for animal experimentation The study protocol was reviewed and approved by the Experimental Research and Skill Development Center (BADABEM), Istanbul Bagcilar Health Application and Research Center, University of Health Sciences (Meeting No.: 2023/35, Project No.: 2023-11). Every effort was made to minimize animal suffering and reduce the number of animals used.

## 3. Results

### 3.1. Biomechanical Evaluation

The maximum load to failure (Fmax) values for the four groups are presented in Figure 2C. Although the mean Fmax was numerically higher in the 30-day subgroups compared with the 15-day subgroups within both the control and Tranilast-treated animals, these differences did not reach statistical significance (*p* = 0.3948).

Biomechanical testing revealed no statistically significant differences in maximum load to failure between control and tranilast-treated tendons at either 15 or 30 days, although a non-significant trend toward higher values was observed at day 30 in both groups.

### 3.2. Bonar Histopathological Scoring

**Figure 3:**
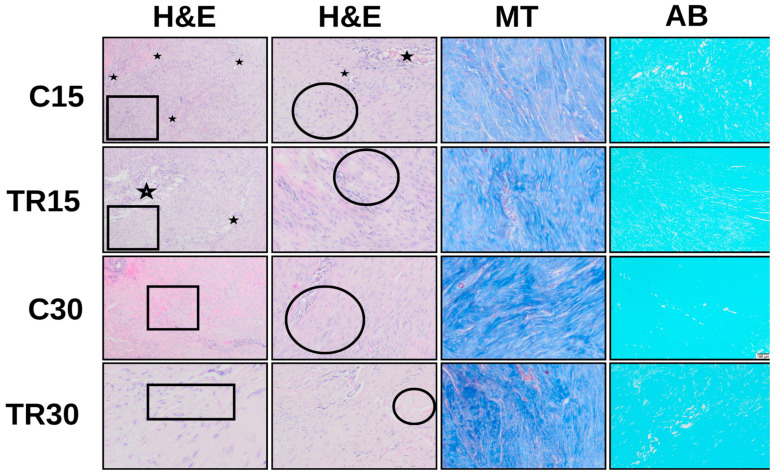
Representative histological images of repaired Achilles tendons from control (C-15, C-30) and tranilast-treated (TR-15, TR-30) groups stained with hematoxylin–eosin (HE), Masson trichrome (MT), and Alcian blue (AB). Square-marked areas in HE images indicate representative regions of cellularity used for semi-quantitative scoring. Asterisks (*) highlight vascular structures corresponding to the vascularity component of the Bonar and Movin scoring systems. Circled regions indicate collagen bundle organization and alignment patterns, which are particularly evident in low-magnification views. Representative images were selected from areas corresponding to the dominant histopathological features used for scoring. At day 15, cellularity and vascularity were more prominent, whereas at day 30, collagen organization and matrix remodeling features predominated. All photomicrographs were acquired at uniform magnification with a 50 μm scale bar to allow for direct comparisons between groups.

**Figure 4:**
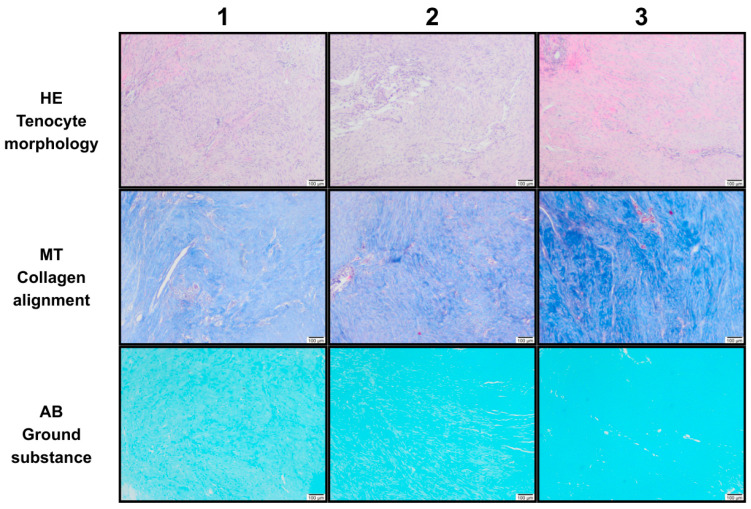
Representative histological images illustrating the morphological features corresponding to increasing Bonar score grades. Images were selected from study specimens and demonstrate characteristic changes in tendon cellularity, collagen bundle organization, vascularity, and ground substance accumulation used during semi-quantitative scoring. Representative regions were chosen to reflect the dominant histopathological features associated with each score category. All images were obtained under identical imaging conditions and magnification.

Significant differences were observed among the four groups across all Bonar scoring parameters (Table 2). Tranilast-treated tendons demonstrated lower overall Bonar scores than controls, with differences mainly involving tenocyte morphology, ground substance (GAG), collagen bundle alignment, and vascularity depending on the specific comparison (Figure 3 and Figure 4). These improvements were evident during both the early reparative/proliferative phase (day 15) and the early remodeling phase (day 30). Consequently, total Bonar scores were significantly lower in the TR-15 and TR-30 groups than in their respective control counterparts, indicating a marked attenuation of degenerative tendon changes with tranilast treatment.

Although vascularity scores were numerically lower in TR-15 than in C-15, this difference did not reach statistical significance, which may reflect the expected peak neovascular response around two weeks after repair and the limited statistical power associated with small subgroup sizes. By day 30, physiological regression of neovessels during the remodeling phase may have accentuated between-group differences, resulting in a statistically significant reduction in vascularity in the TR-30 group compared with C-30.

### 3.3. Movin Histopathological Scoring

Movin scoring demonstrated significant overall differences among groups, reflecting distinct patterns of tendon matrix organization and cellular morphology (Table 3). Compared with controls, tranilast-treated tendons showed lower scores in several Movin domains, including fiber structure, fiber arrangement, nuclear morphology, regional cellularity, vascularity, decreased collagen staining, and hyalinization, although not all pairwise comparisons were significant at both time points. GAG content did not differ significantly between groups. Consequently, total Movin scores were significantly lower in both TR-15 and TR-30 groups than in their respective controls, indicating an overall improvement in histopathological tendon quality with tranilast treatment (Figure 3).

### 3.4. Sirius Red Birefringence Analysis

Sirius Red birefringence analysis revealed no statistically significant differences among groups in collagen type I intensity, type III intensity, or polarization ratios (Table 1). Although numerical variability was observed across time points and treatment groups, none of these differences reached statistical significance. Similarly, the polarization ratio [Type I/(Type I + Type III)], which reflects collagen maturation, remained comparable across all groups. These findings indicate that tranilast did not induce measurable changes in collagen birefringence or fiber-level maturation within the 30-day observation period, despite the clear histological improvements observed in other tendon-healing parameters.

### 3.5. Immunohistochemical Expression (H-Score)

Immunohistochemical assessment of collagen Type I and Type III expression revealed no statistically significant differences among the groups (Table 4). For Collagen Type I, H-scores exhibited numerical variation (*p* = 0.1888). C-15 showed the highest expression (39.00 ± 1.41), while C-30 demonstrated the lowest (11.67 ± 7.64). TR-15 and TR-30 presented intermediate values (22.50 ± 3.54 and 32.50 ± 24.75, respectively). However, none of the pairwise post hoc comparisons reached statistical significance, indicating that Tranilast did not significantly alter Type I collagen immunoreactivity.

**Figure 5:**
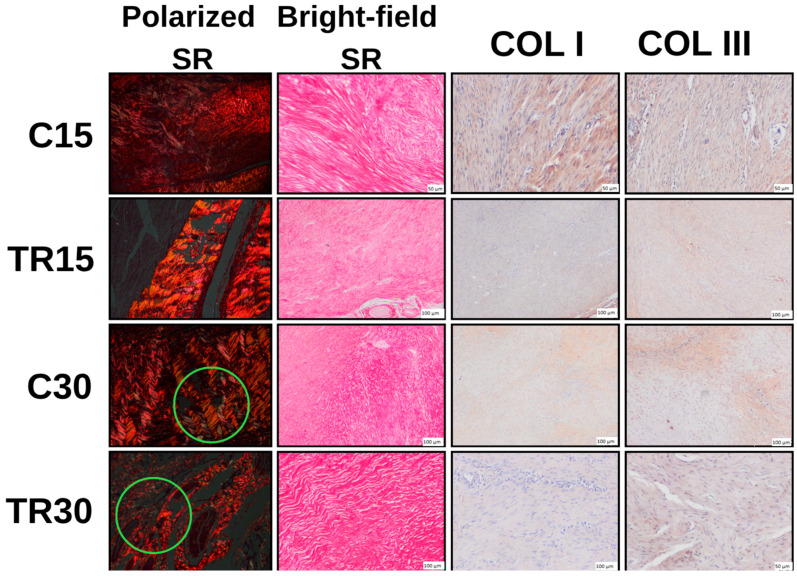
Sirius Red staining and immunohistochemical expression of collagen type I (COL-I) and collagen type III (COL-III) in repaired Achilles tendons from control (C-15, C-30) and tranilast-treated (TR-15, TR-30) groups. Bright-field Sirius Red images demonstrate overall collagen distribution, while polarized light images reveal collagen fiber birefringence patterns corresponding to collagen type I (red–orange) and collagen type III (green–yellow). Polarized light images are presented for qualitative visualization of birefringence patterns only; quantitative collagen measurements were derived from digital bright-field image analysis. Circled areas in selected panels highlight regions exhibiting green birefringence, particularly in the day 30 groups (C-30 and TR-30). COL-I and COL-III immunohistochemical panels demonstrate collagen expression patterns at the repair site. Representative images were selected from regions corresponding to areas used for semi-quantitative analysis. No consistent visual differences in collagen subtype distribution were observed between groups, in agreement with the quantitative findings.

Similarly, Collagen Type III expression did not differ significantly across groups (*p* = 0.2460). Type III H-scores were comparable between C-15 (18.33 ± 2.89), C-30 (13.33 ± 7.64), and TR-15 (16.67 ± 5.77). TR-30 showed a higher mean value (38.33 ± 28.43), but the variation was wide and statistically insignificant (Figure 5).

## 4. Discussion

Tendon healing is a complex, multi-stage process characterized by an early reparative/proliferative phase, followed by proliferative matrix deposition and progressive remodeling [33]. Even with modern surgical repair techniques, the regenerated tissue often remains mechanically inferior to native tendon because healing frequently proceeds through scar-prone remodeling rather than true regeneration [34]. Therefore, pharmacologic agents capable of modulating these pathological features have gained increasing attention [2,4,5,9,35].

In the present study, we evaluated the effects of Tranilast, an antifibrotic and anti-inflammatory agent reported to modulate TGF-β-mediated pathways, in a rat Achilles tendon repair model. Tranilast treatment resulted in significantly lower Bonar and Movin scores during both the reparative/proliferative and early remodeling phases, indicating improved histopathological healing. These improvements were primarily reflected by more favorable tenocyte morphology, collagen organization, and vascularity scores. In contrast, no significant differences were observed in Sirius Red birefringence, immunohistochemical collagen expression, or biomechanical strength. Collectively, these findings suggest that Tranilast is associated with improved histopathological features of tendon healing, although these effects were not accompanied by measurable changes in collagen maturation or mechanical properties within the 30-day observation period. Minor discrepancies between Bonar and Movin GAG-related findings likely reflect differences in scoring criteria and local tissue heterogeneity.

Clinically, an intervention that improves tendon matrix quality (by reducing abnormal cellularity, neovascularization, and matrix degeneration) may be valuable even when early tensile strength is unchanged, because these histological domains are linked to scar-prone healing and tendinopathy-like remodeling. Commonly used adjunctive treatments in clinical practice (e.g., NSAIDs or corticosteroids) primarily target inflammation and pain, yet their effects on tendon structure and long-term mechanical recovery are inconsistent, and excessive suppression of inflammatory signaling may impair matrix remodeling [7,8]. In this context, tranilast may represent a mechanistically distinct strategy aimed at modulating fibrotic pathways and improving tissue organization, although adequately powered studies with longer follow-up are required to determine whether these microscopic benefits translate into clinically meaningful functional gains.

Although tranilast improved histopathological healing, these changes were not accompanied by measurable gains in biomechanical strength. Similar discrepancies between microscopic and mechanical outcomes have been reported in previous studies evaluating biological and pharmacological modulation of tendon healing, suggesting that favorable tissue architecture does not necessarily translate into early improvements in tensile properties [4,5,34].

A similar pattern was reported by Liu et al. (2025), one of the few experimental studies evaluating the biomechanical effects of tranilast in skeletal tissue. Although weight-bearing exercise improved femoral mechanical properties, the addition of tranilast attenuated these gains, suggesting that antifibrotic modulation may influence the tissue remodeling processes required for mechanical reinforcement [36]. Consistent with these findings, tranilast did not improve biomechanical strength in the present study despite favorable histopathological outcomes. However, the relatively small subgroup size and limited follow-up period warrant cautious interpretation of these results. An alternative explanation is that tranilast may primarily modulate scar formation and tissue appearance without substantially enhancing functional tendon regeneration during the early stages of healing. Histopathological improvements, including reduced cellular degeneration, lower vascularity, and improved collagen organization, do not necessarily indicate restoration of the native hierarchical structure required for mechanical load transmission. Therefore, the observed improvements in Bonar and Movin scores may reflect a more mature histological appearance rather than a corresponding increase in functional tissue competence. This distinction may partly explain why significant microscopic improvements were not accompanied by measurable gains in maximum load to failure within the 30-day observation period.

Although no previous study has evaluated tranilast using Bonar or Movin scoring systems, the histopathological improvements observed in our study are consistent with its known anti-inflammatory and antifibrotic properties. Tranilast-treated tendons demonstrated more favorable scores in several domains, including vascularity, collagen organization-related features, nuclear morphology, and hyalinization, suggesting a shift toward a more mature tendon matrix. These findings are in line with previous reports showing that pathological tendon degeneration is characterized by increased vascularity, matrix disorganization, and altered cellular morphology [37], while tranilast has been shown to suppress fibroblast activity and modulate profibrotic signaling pathways, including TGF-β-related mechanisms [35,36,38]. However, because molecular markers were not directly assessed in the present study, the involvement of these pathways remains speculative and warrants further investigation. Furthermore, neither Sirius Red birefringence analysis nor collagen type I/III immunohistochemical expression demonstrated significant differences between groups. Therefore, the biological mechanisms underlying the observed histopathological improvements remain unresolved, and these findings should not be interpreted as direct evidence of antifibrotic activity within the healing tendon. Future studies evaluating fibrosis-related molecular markers, including TGF-β1, CTGF, α-SMA, COL1A1, and COL3A1, are required to clarify the pathways responsible for these observations.

The reduction in hyalinization further supports a potential modulatory effect of tranilast on tendon remodeling. Previous studies in fibrotic disease models have shown that tranilast can attenuate pathological tissue remodeling and influence extracellular matrix homeostasis [39,40]. Consistent with these observations, tranilast-treated tendons in the present study exhibited lower Bonar and Movin scores, suggesting improved histopathological remodeling.

In our study, Sirius Red birefringence and collagen type I/III immunohistochemical expression did not differ significantly between groups at either time point, suggesting that tranilast did not substantially alter collagen composition during the early phases of tendon healing. These methods are semi-quantitative and may be influenced by fiber orientation, staining conditions, and tissue heterogeneity, which could contribute to variability in collagen-related measurements. Although previous experimental studies have reported antifibrotic effects of tranilast, including suppression of collagen synthesis and extracellular matrix remodeling [17,22,41,42], such effects were not clearly demonstrated in the present tendon repair model. This discrepancy may indicate that tranilast influences tissue organization and remodeling more readily than overall collagen content during early healing [43].

This study has several limitations. First, the model represents acute tendon injury and repair rather than chronic degenerative tendinopathy, which may limit direct clinical translation. Second, the follow-up period was restricted to 15 and 30 days and may not have captured long-term effects of tranilast on tendon remodeling and mechanical maturation. Because only the reparative/proliferative and early remodeling phases were evaluated, potential effects of tranilast on the initial inflammatory response, including neutrophil and macrophage infiltration during the first postoperative week, could not be assessed [44]. Furthermore, biomechanical assessment was limited to maximum load to failure. Additional mechanical parameters, including stiffness, viscoelastic behavior, and other functional properties evaluated in previous tendon-healing studies, may provide complementary information regarding tissue quality and functional recovery [45]. Although tranilast produced favorable histopathological changes, no significant biomechanical differences were observed; this may reflect the limited follow-up period and the relatively small biomechanical sample size (n = 4 per subgroup), increasing the risk of type II errors. Residual suture material may have also influenced tensile testing results. The biomechanical subgroup size was small (n = 4 per group). Sensitivity-based power estimation indicated that this sample size would only detect very large effects in maximum load to failure; therefore, smaller but potentially meaningful treatment-related biomechanical differences may have remained undetected. Third, histological assessment was performed by a single blinded evaluator, and inter-observer reliability was not assessed. Finally, Sirius Red birefringence and immunohistochemical analyses are semi-quantitative methods and may not detect subtle alterations in collagen organization or turnover. Furthermore, detailed methodological records regarding the immunohistochemical evaluation, including the exact number of microscopic fields analyzed per specimen, magnification used for scoring, intensity-grading procedures, and the use of software-assisted image analysis, were not available because the assessment was performed as part of routine pathological evaluation. Consequently, the reproducibility of this aspect of the study is limited and the immunohistochemical findings should be interpreted with appropriate caution. Future studies incorporating longer follow-up, larger sample sizes, quantitative molecular analyses, and automated image-based assessment techniques are warranted [46].

Importantly, the favorable findings observed in the tranilast-treated groups should be interpreted as improvements in histopathological appearance rather than definitive enhancement of tendon regeneration. Although Bonar and Movin scores indicated more mature tissue architecture and improved matrix organization, no significant differences were detected in collagen subtype expression, collagen birefringence, or biomechanical strength. Therefore, restoration of native tendon structure and function cannot be concluded from the present data. Furthermore, ultrastructural assessment of collagen fibril architecture and fiber orientation was not performed; consequently, conclusions regarding collagen alignment and matrix remodeling are based on established histopathological scoring systems rather than direct ultrastructural evaluation. Future studies incorporating advanced imaging techniques, molecular analyses, and longer-term functional assessment are required to clarify the biological and functional significance of these findings.

Furthermore, the Bonar and Movin scoring systems were originally developed for the assessment of chronic tendinopathic tissue rather than acute tendon healing. Although both systems are widely used in experimental tendon-healing studies and provide standardized semi-quantitative assessment of tissue architecture, cellularity, vascularity, and extracellular matrix organization, their application to regenerative tendon repair should be interpreted with caution.

Additionally, advanced quantitative morphometric analyses of collagen architecture and proteoglycan distribution were not performed. Future studies incorporating objective digital image-analysis techniques and automated quantitative assessment methods may help further validate the semi-quantitative histological findings reported here.

Tranilast was administered systemically rather than locally, and drug exposure at the repair site was not assessed. Therefore, the optimal route of administration and dose for tendon healing remain uncertain. In addition, functional outcome measures such as gait analysis or grip-strength testing were not evaluated. Future studies incorporating local delivery strategies, functional assessments, and longer follow-up periods may provide a more comprehensive evaluation of tendon healing.

## 5. Conclusions

In this experimental rat model of Achilles tendon repair, tranilast was associated with favorable histopathological changes, reflected by lower Bonar and Movin scores and improvements in several microscopic features of tendon healing. However, these findings should be considered preliminary, as no corresponding differences were observed in collagen birefringence, collagen type I/III expression, or biomechanical strength during the 30-day follow-up period. Collectively, the results suggest that tranilast may influence histopathological remodeling during tendon healing, although the underlying mechanisms and functional significance remain uncertain. Further studies incorporating longer follow-up periods, molecular analyses, functional outcome measures, and alternative delivery strategies are required to clarify the potential role of tranilast in tendon repair.

## Figures and Tables

**Table 1:** Sirius Red birefringence analysis (Mean ± SD).

Parameter	C-15	C-30	TR-15	TR-30	*p* (Overall)	Post Hoc *p* Values	
**Mean Type I intensity (0–3)**	2.63 ± 0.25	1.88 ± 0.63	1.89 ± 0.63	3.00 ± 1.41	0.2103	C-15 vs. C-30	0.6548
						C-15 vs. TR-15	0.6548
						C-15 vs. TR-30	0.7904
						C-30 vs. TR-15	0.9837
						C-30 vs. TR-30	0.4105
						TR-15 vs. TR-30	0.4105
**Mean Type III intensity (0–3)**	1.25 ± 0.29	1.00 ± 0.00	0.99 ± 0.39	1.50 ± 0.58	0.2279	C-15 vs. C-30	0.8145
						C-15 vs. TR-15	0.8145
						C-15 vs. TR-30	0.8145
						C-30 vs. TR-15	0.9633
						C-30 vs. TR-30	0.3870
						TR-15 vs. TR-30	0.3870
**Polarization ratio = Type I/(Type I + Type III)**	0.68 ± 0.07	0.64 ± 0.09	0.65 ± 0.14	0.61 ± 0.08	0.3001	C-15 vs. C-30	0.9865
						C-15 vs. TR-15	0.9865
						C-15 vs. TR-30	0.9375
						C-30 vs. TR-15	0.9865
						C-30 vs. TR-30	0.9865
						TR-15 vs. TR-30	0.9865

Quantitative values were derived from digital bright-field image analysis; polarized light microscopy was used for qualitative assessment only.

**Table 2:** Bonar histopathological scoring (Mean ± SD).

Parameter	C-15	C-30	TR-15	TR-30	*p* (Overall)	Post Hoc *p* Value	
**Tenocyte morphology/proliferation (0–3)**	3.00 ± 0.00	2.50 ± 0.58	1.00 ± 0.00	1.50 ± 0.58	**0.0005**	C-15 vs. C-30	>0.9999
						C-15 vs. TR-15	**0.0094**
						C-15 vs. TR-30	0.1062
						C-30 vs. TR-15	0.1062
						C-30 vs. TR-30	0.6831
						TR-15 vs. TR-30	>0.9999
**Ground substance/GAG (0–3)**	2.50 ± 0.58	2.00 ± 0.00	0.25 ± 0.50	0.50 ± 0.58	**0.0002**	C-15 vs. C-30	>0.9999
						C-15 vs. TR-15	**0.0187**
						C-15 vs. TR-30	**0.0491**
						C-30 vs. TR-15	0.1178
						C-30 vs. TR-30	0.2590
						TR-15 vs. TR-30	>0.9999
**Collagen bundle alignment (0–3)**	2.75 ± 0.50	2.50 ± 0.58	0.75 ± 0.50	1.50 ± 0.58	**0.0023**	C-15 vs. C-30	>0.9999
						C-15 vs. TR-15	**0.0187**
						C-15 vs. TR-30	0.3112
						C-30 vs. TR-15	0.0616
						C-30 vs. TR-30	0.7191
						TR-15 vs. TR-30	>0.9999
**Vascularity (0–3)**	2.00 ± 0.00	2.00 ± 0.00	1.00 ± 0.00	0.50 ± 0.58	**0.0002**	C-15 vs. C-30	>0.9999
						C-15 vs. TR-15	0.1317
						C-15 vs. TR-30	**0.0193**
						C-30 vs. TR-15	0.1317
						C-30 vs. TR-30	**0.0193**
						TR-15 vs. TR-30	>0.9999
**TOTAL BONAR (0–12)**	10.25 ± 0.96	9.00 ± 1.16	3.00 ± 0.82	4.00 ± 2.31	**<0.0001**	C-15 vs. C-30	0.4253
						C-15 vs. TR-15	**<0.0001**
						C-15 vs. TR-30	**0.0002**
						C-30 vs. TR-15	**0.0003**
						C-30 vs. TR-30	**0.0011**
						TR-15 vs. TR-30	0.4253

**Table 3:** Movin histopathological scoring (Mean ± SD).

Parameter	C-15	C-30	TR-15	TR-30	*p* (Overall)	Post Hoc *p* Value	
**Fiber structure (0–3)**	2.75 ± 0.50	2.50 ± 0.58	0.75 ± 0.50	1.25 ± 0.96	**0.0027**	C-15 vs. C-30	0.6027
						C-15 vs. TR-15	**0.0064**
						C-15 vs. TR-30	**0.0298**
						C-30 vs. TR-15	**0.0140**
						C-30 vs. TR-30	0.0597
						TR-15 vs. TR-30	0.5185
**Fiber arrangement (0–3)**	2.75 ± 0.50	2.50 ± 0.58	1.00 ± 0.00	1.50 ± 0.58	**0.0007**	C-15 vs. C-30	0.4744
						C-15 vs. TR-15	**0.0014**
						C-15 vs. TR-30	**0.0122**
						C-30 vs. TR-15	**0.0041**
						C-30 vs. TR-30	**0.0357**
						TR-15 vs. TR-30	0.3035
**Nuclear morphology (0–3)**	3.00 ± 0.00	2.25 ± 0.50	1.00 ± 0.00	1.00 ± 0.00	**<0.0001**	C-15 vs. C-30	**0.0023**
						C-15 vs. TR-15	**<0.0001**
						C-15 vs. TR-30	**<0.0001**
						C-30 vs. TR-15	**<0.0001**
						C-30 vs. TR-30	**<0.0001**
						TR-15 vs. TR-30	>0.9999
**Regional cellularity (0–3)**	2.00 ± 0.00	2.00 ± 0.00	0.75 ± 0.50	1.00 ± 0.00	**<0.0001**	C-15 vs. C-30	>0.9999
						C-15 vs. TR-15	**<0.0001**
						C-15 vs. TR-30	**0.0004**
						C-30 vs. TR-15	**<0.0001**
						C-30 vs. TR-30	**0.0004**
						TR-15 vs. TR-30	0.3320
**Increased vascularity (0–3)**	2.00 ± 0.00	2.50 ± 0.58	0.25 ± 0.50	0.50 ± 0.58	**<0.0001**	C-15 vs. C-30	0.3035
						C-15 vs. TR-15	**0.0009**
						C-15 vs. TR-30	**0.0025**
						C-30 vs. TR-15	**0.0001**
						C-30 vs. TR-30	**0.0004**
						TR-15 vs. TR-30	0.4744
**Decreased collagen staining (MT) (0–3)**	2.50 ± 0.58	2.00 ± 0.00	0.75 ± 0.50	0.75 ± 0.50	**0.0002**	C-15 vs. C-30	0.2729
						C-15 vs. TR-15	**0.0009**
						C-15 vs. TR-30	**0.0009**
						C-30 vs. TR-15	**0.0088**
						C-30 vs. TR-30	**0.0088**
						TR-15 vs. TR-30	>0.9999
**Hyalinization (0–3)**	1.00 ± 0.00	1.00 ± 0.00	0.00 ± 0.00	0.50 ± 0.58	**0.0009**	C-15 vs. C-30	>0.9999
						C-15 vs. TR-15	**0.0022**
						C-15 vs. TR-30	0.1170
						C-30 vs. TR-15	**0.0022**
						C-30 vs. TR-30	0.1170
						TR-15 vs. TR-30	0.1170
**GAG content (AB) (0–3)**	2.50 ± 0.58	2.00 ± 0.00	2.00 ± 2.71	0.50 ± 0.58	0.2646	C-15 vs. C-30	0.9477
						C-15 vs. TR-15	0.9477
						C-15 vs. TR-30	0.3474
						C-30 vs. TR-15	>0.9999
						C-30 vs. TR-30	0.5804
						TR-15 vs. TR-30	0.5804
**TOTAL MOVIN (0–24)**	18.50 ± 1.92	17.50 ± 2.65	7.25 ± 1.50	7.50 ± 4.12	**<0.0001**	C-15 vs. C-30	0.8514
						C-15 vs. TR-15	**0.0005**
						C-15 vs. TR-30	**0.0005**
						C-30 vs. TR-15	**0.0008**
						C-30 vs. TR-30	**0.0008**
						TR-15 vs. TR-30	0.8993

**Table 4:** Immunohistochemical expression (H-Score) (Mean ± SD).

Parameter	C-15	C-30	TR-15	TR-30	*p* (Overall)	Post Hoc *p* Value	
**Collagen Type I (H-Score, 0–300)**	39.00 ± 1.41	11.67 ± 7.64	22.50 ± 3.54	32.50 ± 24.75	0.1888	C-15 vs. C-30	0.2994
						C-15 vs. TR-15	0.6558
						C-15 vs. TR-30	0.7561
						C-30 vs. TR-15	0.7561
						C-30 vs. TR-30	0.4729
						TR-15 vs. TR-30	0.7561
**Collagen Type III (H-Score, 0–300)**	18.33 ± 2.89	13.33 ± 7.64	16.67 ± 5.77	38.33 ± 28.43	0.2460	C-15 vs. C-30	0.9717
						C-15 vs. TR-15	0.9717
						C-15 vs. TR-30	0.4610
						C-30 vs. TR-15	0.9717
						C-30 vs. TR-30	0.3802
						TR-15 vs. TR-30	0.4610

## Data Availability

All data generated or analyzed during this study are included in this article. Data are available on request due to privacy/ethical restrictions.

## References

[1] Sharma P., Maffulli N. (2006). Biology of tendon injury: Healing, modeling and remodeling. J. Musculoskelet. Neuronal Interact..

[2] Maffulli N., Cuozzo F., Migliorini F., Oliva F. (2023). The tendon unit: Biochemical, biomechanical, hormonal influences. J. Orthop. Surg. Res..

[3] Nichols A.E.C., Best K.T., Loiselle A.E. (2019). The cellular basis of fibrotic tendon healing: Challenges and opportunities. Transl. Res..

[4] Citeroni M.R., Ciardulli M.C., Russo V., Della Porta G., Mauro A., El Khatib M., Di Mattia M., Galesso D., Barbera C., Forsyth N.R. (2020). In Vitro Innovation of Tendon Tissue Engineering Strategies. Int. J. Mol. Sci..

[5] Migliorini F., Tingart M., Maffulli N. (2020). Progress with stem cell therapies for tendon tissue regeneration. Expert Opin. Biol. Ther..

[6] Swiderski K., Todorov M., Gehrig S.M., Naim T., Chee A., Stapleton D.I., Koopman R., Lynch G.S. (2014). Tranilast administration reduces fibrosis and improves fatigue resistance in muscles of mdx dystrophic mice. Fibrogenesis Tissue Repair.

[7] Longo U.G., Lamberti A., Maffulli N., Denaro V. (2011). Tissue engineered biological augmentation for tendon healing: A systematic review. Br. Med. Bull..

[8] Citro V., Clerici M., Boccaccini A.R., Della Porta G., Maffulli N., Forsyth N.R. (2023). Tendon tissue engineering: An overview of biologics to promote tendon healing and repair. J. Tissue Eng..

[9] Dale T.P., Mazher S., Webb W.R., Zhou J., Maffulli N., Chen G.-Q., El Haj A.J., Forsyth N.R. (2018). Tenogenic Differentiation of Human Embryonic Stem Cells. Tissue Eng. Part. A.

[10] García Mesa M. (1990). New approach to the mechanism of antiasthmatic action of Tranilast. Allergol. Immunopathol. (Madr.).

[11] Kim S.J., Kim J.W., Kim Y.H., Lee S.H., Yoon H.K., Kim C.H., Ahn J.H., Lee J.M., Kim J.S., Kim S.C. (2009). Effects of tranilast and pentoxifylline in a mouse model of chronic asthma using house dust mite antigen. J. Asthma.

[12] Kondo N., Fukutomi O., Kameyama T., Orii T. (1992). Inhibition of proliferative responses of lymphocytes to food antigens by an anti-allergic drug, N(3’,4’-dimethoxycinnamoyl) anthranilic acid (Tranilast) in children with atopic dermatitis. Clin. Exp. Allergy.

[13] Kondo N., Fukutomi O., Shinbara M., Orii T. (1994). Inhibition of interferon-gamma and interleukin-2 production from lymphocytes stimulated with food antigens by an anti-allergic drug, Tranilast, in patients with food-sensitive atopic dermatitis. Biotherapy.

[14] Shimizu T., Kanai K., Asano K., Hisamitsu T., Suzaki H. (2005). Suppression of matrix metalloproteinase production in nasal fibroblasts by tranilast, an antiallergic agent, in vitro. Mediat. Inflamm..

[15] Suzuki H., Tanaka K., Kaneshige H., Nakagami K., Noguchi E. (1989). The effects of long term Tranilast administration on bronchial hypersensitivity in asthmatics. Panminerva Med..

[16] Rogosnitzky M., Danks R., Kardash E. (2012). Therapeutic potential of tranilast, an anti-allergy drug, in proliferative disorders. Anticancer. Res..

[17] Suzawa H., Kikuchi S., Ichikawa K., Koda A. (1992). Inhibitory action of tranilast, an anti-allergic drug, on the release of cytokines and PGE2 from human monocytes-macrophages. Jpn. J. Pharmacol..

[18] Martin J., Kelly D., Mifsud S., Zhang Y., Cox A., See F., Krum H., Wilkinsonberka J., Gilbert R. (2005). Tranilast attenuates cardiac matrix deposition in experimental diabetes: Role of transforming growth factor-beta. Cardiovasc. Res..

[19] Mifsud S., Kelly D.J., Qi W., Zhang Y., Pollock C.A., Wilkinson-Berka J.L., Gilbert R.E. (2003). Intervention with tranilast attenuates renal pathology and albuminuria in advanced experimental diabetic nephropathy. Nephron Physiol..

[20] Miyajima A., Asano T., Asano T., Yoshimura I., Seta K., Hayakawa M. (2001). Tranilast ameliorates renal tubular damage in unilateral ureteral obstruction. J. Urol..

[21] Miyazawa K., Kikuchi S., Fukuyama J., Hamano S., Ujiie A. (1995). Inhibition of PDGF- and TGF-beta 1-induced collagen synthesis, migration and proliferation by tranilast in vascular smooth muscle cells from spontaneously hypertensive rats. Atherosclerosis.

[22] Yamada H., Tajima S., Nishikawa T., Murad S., Pinnell S.R. (1994). Tranilast, a selective inhibitor of collagen synthesis in human skin fibroblasts. J. Biochem..

[23] Iwata Y., Katanosaka Y., Shijun Z., Kobayashi Y., Hanada H., Shigekawa M., Wakabayashi S. (2005). Protective effects of Ca2+ handling drugs against abnormal Ca2+ homeostasis and cell damage in myopathic skeletal muscle cells. Biochem. Pharmacol..

[24] Darakhshan S., Pour A.B. (2015). Tranilast: A review of its therapeutic applications. Pharmacol. Res..

[25] Liu Y., Kan M., Li A., Hou L., Jia H., Xin Y., Liu Y. (2016). Inhibitory Effects of Tranilast on Cytokine, Chemokine, Adhesion Molecule, and Matrix Metalloproteinase Expression in Human Corneal Fibroblasts Exposed to Poly(I:C). Curr. Eye Res..

[26] Maffulli N., Irwin A.S., Kenward M.G., Smith F., Porter R.W. (1998). Achilles tendon rupture and sciatica: A possible correlation. Br. J. Sports Med..

[27] Maffulli N. (1998). Current concepts in the management of subcutaneous tears of the Achilles tendon. Bull. Hosp. Jt. Dis..

[28] Godoy-Santos A.L., Bruschini H., Cury J., Srougi M., de Cesar-Netto C., Fonseca L.F., Maffulli N. (2018). Fluoroquinolones and the Risk of Achilles Tendon Disorders: Update on a Neglected Complication. Urology.

[29] Maffulli N., Christidis G., Gougoulias N., Christidis P., Poku D., Hassan R., Migliorini F., Oliva F. (2025). Percutaneous repair of the Achilles tendon with one knot offers equivalent results as the same procedure with two knots. A comparative prospective study. Br. Med. Bull..

[30] Iborra A., Villanueva M., Ferro D., Palacios J., Maffulli N. (2025). Percutaneous ultrasound-guided repair of Achilles tendon rupture: A cadaveric study and preliminary clinical report. J. Orthop. Surg. Res..

[31] Saxena T., Saxena A., Royds M., Maffulli N. (2026). Acute Achilles tendon rupture and chronic tendinopathy surgery: Same tendon, with sex and ethnicity differences. Foot Ankle Surg..

[32] European Commission (2010). Directive 2010/63/EU of the European Parliament and of the Council of 22 September 2010 on the protection of animals used for scientific purposes. Off. J. Eur. Union.

[33] Gonçalves A.I., Righelli L., Reis R.L., El Haj A.J., Gomes M.E. (2025). Understanding Degeneration and Healing Pathways for Tissue-Engineered Treatment Strategies in Tendinopathy. Cells Tissues Organs.

[34] Graham J.G., Wang M.L., Rivlin M., Beredjiklian P.K. (2019). Biologic and mechanical aspects of tendon fibrosis after injury and repair. Connect. Tissue Res..

[35] Najafi Z., Rahmanian-Devin P., Baradaran Rahimi V., Nokhodchi A., Askari V.R. (2024). Challenges and opportunities of medicines for treating tendon inflammation and fibrosis: A comprehensive and mechanistic review. Fundam. Clin. Pharmacol..

[36] Liu M., Han Y., Wang J., Zhu Y., Zhang Y., Chu Q., Yang C., Chen B., Sun G. (2025). Skeletal muscle-derived IL-33 mediates muscle-to-bone crosstalk and regulates bone metabolism via CD8(+) T cell-secreted CCL5. EBioMedicine.

[37] Kelly D.J., Zhang Y., Connelly K., Cox A.J., Martin J., Krum H., Gilbert R.E. (2007). Tranilast attenuates diastolic dysfunction and structural injury in experimental diabetic cardiomyopathy. Am. J. Physiol. Heart Circ. Physiol..

[38] Han C., Li X., Zhou T., Chen C., Zhang K., Yang S., Wang X., Tian H., Zhao C., Zhao J. (2019). A tranilast and BMP-2 based functional bilayer membrane is effective for the prevention of epidural fibrosis during spinal lamina reconstruction. J. Mater. Chem. B.

[39] Chen S., Wang Y., Pan Y., Liu Y., Zheng S., Ding K., Mu K., Yuan Y., Li Z., Song H. (2020). Novel Role for Tranilast in Regulating NLRP3 Ubiquitination, Vascular Inflammation, and Atherosclerosis. J. Am. Heart Assoc..

[40] Massoud G., Parish M., Hazimeh D., Moukarzel P., Singh B., Vaught K.C.C., Segars J., Islam S. (2024). Unlocking the potential of tranilast: Targeting fibrotic signaling pathways for therapeutic benefit. Int. Immunopharmacol..

[41] Isaji M., Aruga N., Naito J., Miyata H. (1994). Inhibition by tranilast of collagen accumulation in hypersensitive granulomatous inflammation in vivo and of morphological changes and functions of fibroblasts in vitro. Life Sci..

[42] Liu Y., Zhao X.-J., Zheng X.-S., Zheng H., Liu L., Meng L.-B., Li Q., Liu Y. (2018). Tranilast inhibits TGF-β-induced collagen gel contraction mediated by human corneal fibroblasts. Int. J. Ophthalmol..

[43] Gajhede-Knudsen M., Ekstrand J., Magnusson H., Maffulli N. (2013). Recurrence of Achilles tendon injuries in elite male football players is more common after early return to play: An 11-year follow-up of the UEFA Champions League injury study. Br. J. Sports Med..

[44] Cheng S., Yang J., Song J., Cao X., Zhou B., Yang L., Li C., Wang Y. (2025). A motion-responsive injectable lubricative hydrogel for Efficient Achilles tendon adhesion prevention. Mater. Today Bio.

[45] Bi C., Thoreson A.R., Zhao C. (2023). Improving Mechanical Properties of Tendon Allograft through Rehydration Strategies: An In Vitro Study. Bioengineering.

[46] DeFoor M.T., Cognetti D.J., Yuan T.T., Sheean A.J. (2024). Treatment of Tendon Injuries in the Servicemember Population across the Spectrum of Pathology: From Exosomes to Bioinductive Scaffolds. Bioengineering.

